# In vitro inhibitory effect of obtusofolin on the activity of CYP3A4, 2C9, and 2E1

**DOI:** 10.1186/s12906-021-03397-w

**Published:** 2021-09-01

**Authors:** Na Liu, Ping Chen, Xiaojun Du, Junxia Sun, Shasha Han

**Affiliations:** 1 Department of Ophthalmology, Dongying People’s Hospital, No. 317, Nanyi Road, Dongcheng, Dongying, 257091 Shandong Province China; 2 Department of Ophthalmology, Shengli Oilfield Central Hospital, Dongying, 257034 Shandong China

**Keywords:** Obtusofolin, Cytochrome P450 enzymes, Dose-dependent, Time-dependent, Drug-drug interaction

## Abstract

**Background:**

Obtusofolin is the major active ingredient of Catsia tora L., which possesses the activity of improving eyesight and protecting the optic nerve. Investigation on the interaction of obtusofolin with cytochrome P450 enzymes (CYP450s) could provide a reference for the clinical application of obtusofolin.

**Methods:**

The effect of obtusofolin on the activity of CYP450s was investigated in the presence of 100 μM obtusofolin in pooled human liver microsomes (HLMs) and fitted with the Lineweaver–Burk plots to characterize the specific inhibition model and kinetic parameters.

**Results:**

Obtusofolin was found to significantly inhibited the activity of CYP3A4, 2C9, and 2E1. In the presence of 0, 2.5, 5, 10, 25, 50, and 100 μM obtusofolin, the inhibition of these CYP450s showed a dose-dependent manner with the IC_50_ values of 17.1 ± 0.25, 10.8 ± 0.13, and 15.5 ± 0.16 μM, respectively. The inhibition of CYP3A4 was best fitted with the non-competitive inhibition model with the *K*_*i*_ value of 8.82 μM. While the inhibition of CYP2C9 and 2E1 was competitive with the *K*_*i*_ values of 5.54 and 7.79 μM, respectively. After incubating for 0, 5, 10, 15, and 30 min, the inhibition of CYP3A4 was revealed to be time-dependent with the *K*_*I*_ value of 4.87 μM^− 1^ and the *K*_*inact*_ value of 0.0515 min^− 1^.

**Conclusions:**

The in vitro inhibitory effect of obtusofolin implying the potential drug-drug interaction between obtusofolin and corresponding substrates, which needs further in vivo validations.

## Introduction

*Catsia tora Linn* can be used as a food or medicine in traditional Chinese medicine, which possesses the activity of improving eyesight and protecting the optic nerve [1]. Obtusofolin is the major active ingredient of *Catsia tora L.* and has the effect of anti-oxidation [2]. The clinical significance of obtusofolin also includes alleviating hyperlipidemia and hyperglycemia, against inflammatory and neuropathic pain, and ameliorate memory impairment [3–5]. In ophthalmology, obtusofolin could attenuate the apoptosis of retinal capillary cells and suppress the development of retinopathy induced by diabetes [6]. It has been reported that obtusofolin could inhibit the growth of retinal pigment epithelial cells under hypoxia and therefore suppressed the pathological basis of angiogenesis [7].

Cytochrome P450 enzymes (CYP450s) are a series of membrane-bound hemoproteins that participate in cellular metabolism and the biotransformation of numerous xenobiotics [8]. The activity of CYP450s is a critical factor that may induce unfavorable interactions between various drugs. For example, cannabis has been demonstrated to interact with a variety of drugs, such as warfarin, because of its inhibitory effect on the activity of CYP2C19 [9]. Therefore, it is of great importance to evaluate the effect of different xenobiotics on the activity of major CYP450 isoforms. The clinical application of *C. tora Linn* is getting wider, and the co-administration of obtusofolin and other drugs is a common medication in the clinic [10]. Its effect on the activity of CYP450s could guide the co-administration of obtusofolin and other drugs or herbs in one prescription.

The interaction between obtusofolin and eight major CYP450s (including CYP1A2, 2A6, 3A4, 2C8, 2C9, 2C19, 2D6, and 2E1) was estimated in human liver microsomes in the present study, in order to disclose the effect of obtusofolin on the activity of CYP450s and provide a reference for the clinical use of obtusofolin.

## Materials and methods

The investigation was conducted in pool human liver microsomes in vitro. The activity of CYP450s was evaluated by the specific substrates and marker reactions as previously reported summarized in Table 1 [11, 12]. Except for the HLMs treated with 100 μM obtusofolin, specific inhibitors, and negative control HLMs were also employed.

**Table 1:** Isoforms tested, marker reactions, incubation conditions, and K_m_ used in the inhibition study

CYPs	Marker reactions	Substrate concentration (μM)	Protein concentration (mg/mL)	Incubation time (min)	Estimated K_m_ (μM)	Inhibitors (μM)
1A2	phenacetin O-deethylation	40	0.2	30	48	10 μM furafylline
2A6	coumarin 7-hydroxylation	1.0	0.1	10	1.5	10 μM tranylcypromine
3A4	testosterone 6β-hydroxylation	50	0.5	10	53	1 μM ketoconazole
2C8	paclitaxel 6α-hydroxylation	10	0.5	30	16	5 μM montelukast
2C9	diclofenac 4′-hydroxylation	10	0.3	10	13	10 μM sulphaphenazole
2C19	S-Mephenytoin 4-hydroxylation	100	0.2	40	105	50 μM tranylcypromine
2D6	dextromethorphan O-demethylation	25	0.25	20	4.8	10 μM quinidine
2E1	chlorzoxazone 6-hydroxylation	120	0.4	30	126	50 μM clomethiazole

### Reagents

Obtusofolin was obtained from Chengdu Must Bio-Technology Co. (Chengdu, China). The required substrates and reagents were purchased from Sigma Chemical Co. (Chicago, USA). Pooled HLMs were obtained from BD Bioscience (Woburn, USA). The purity of used chemicals was over 98% and the other reagents were of at least analytical reagent grade.

### Assay with human liver microsomes

The incubation volume was 200 μL containing potassium phosphate buffer (pH 7.4), an NADPH-generating system composed of NADP^+^, glucose-6-phosphate, glucose-6-phosphate dehydrogenase, and MgCl_2_, probe substrates, HLMs, and obtusofolin or positive inhibitors. The concentration of positive inhibitors and microsome proteins were summarized in Table 1. The experiments for each CYP isoforms were performed in triplicate and the obtained results were represented as mean value ± SD.

The reactions were initiated by adding the NADPH-generating system after a 3-min preincubation at 37 °C. The reactions were terminated by adding 100 μL acetonitrile or 10% (v:v) trichloroacetic. The mixture was placed on ice and centrifuged at 12,000 rpm for 10 min. A total of 20 μL supernatant was obtained for the analysis of metabolites by HPLC.

### Enzyme inhibition and dose-dependent experiments

Firstly, 100 μM obtusofolin was incubated with the above incubation system in HLMs to evaluate its effect on the activity of CYP450s. Then the dose-dependent experiments were performed to obtain corresponding parameters in the presence of 0, 2.5, 5, 10, 25, 50, and 100 μM obtusofolin. The concentrations of probe substrates were 20–100 μM testosterone for CYP3A4, 5–20 μM diclofenac for CYP2C9, and 25–250 μM chlorzoxazone for CYP2E1.

### Time-dependent inhibition experiments

The time-dependent inhibition experiments were conducted at the incubation time of 0, 5, 10, 15, and 30 min at 37 °C with 20 μM obtusofolin. After the incubation, a total of 20 μL aliquot was transferred to another tube with the NADPH-generating system and probe substrates and incubation for a specific time. The reactions were terminated by the addition of acetonitrile and placed on ice for further analysis.

The time-dependent characteristic was estimated by the values of *K*_*I*_ and *K*_*inact*_ obtained from further experiments. The incubation was performed with 0, 2, 5, 10, 20, and 50 μM obtusofolin and a higher concentration of substrates (approximately 4-fold to Km) for 0, 5, 10, 15, and 30 min. The incubation scheme was performed as described above. The fitting equation to obtain the value of *K*_*I*_ and *K*_*inact*_ was:

$$ 1/\mathrm{Kobs}={K}_I/{K}_{inact}\ast 1/\left[\mathrm{I}\right]+1/{K}_{inact} $$

where Kobs is the pseudo-first-order rate constant of inactivation at inactivated concentration [I], *K*_*inact*_ is the maximum inactivation rate (a theoretical value that cannot be experimentally observed), and *K*_*I*_ is the inactivated concentration when the rate of inactivation reaches half of *K*_*inact*_.

### Statistical analysis

The enzyme kinetic parameters were obtained by the least-squares linear regression. The inhibition data were fitted with non-linear regression according to the following equation:

$$ \mathrm{V}=\left({V}_{max}S\right)/\left({K}_m\left(1+I/{K}_i\right)+S\right)\ \mathrm{for}\ \mathrm{competitive}\ \mathrm{inhibition}\ \left(\mathrm{CYP}2\mathrm{C}9\ \mathrm{and}\ 2\mathrm{E}1\right); $$

$$ \mathrm{V}=\left({V}_{max}S\right)/\left[{K}_m+S\left(1+I/{K}_i\right)\right]\ \mathrm{for}\ \mathrm{non}-\mathrm{competitive}\ \mathrm{inhibition}\ \left(\mathrm{CYP}3\mathrm{A}4\right). $$

where *I* is the concentration of the compound, *K*_*i*_ is the inhibition constant, *S* is the concentration of the substrate and *K*_*m*_ is the substrate concentration at half the maximum velocity (*V*_*max*_) of the reaction. The mechanism of the inhibition was inspected using the Lineweaver–Burk plots and the enzyme inhibition models. The data comparison was performed using the Student’s t-test and performed using IBM SPSS statistics 20 (SPSS Inc., Chicago, IL, USA).

## Results

### Obtusofolin significantly inhibited the activity of CYP3A4, 2C9, and 2E1

**Fig. 1:**
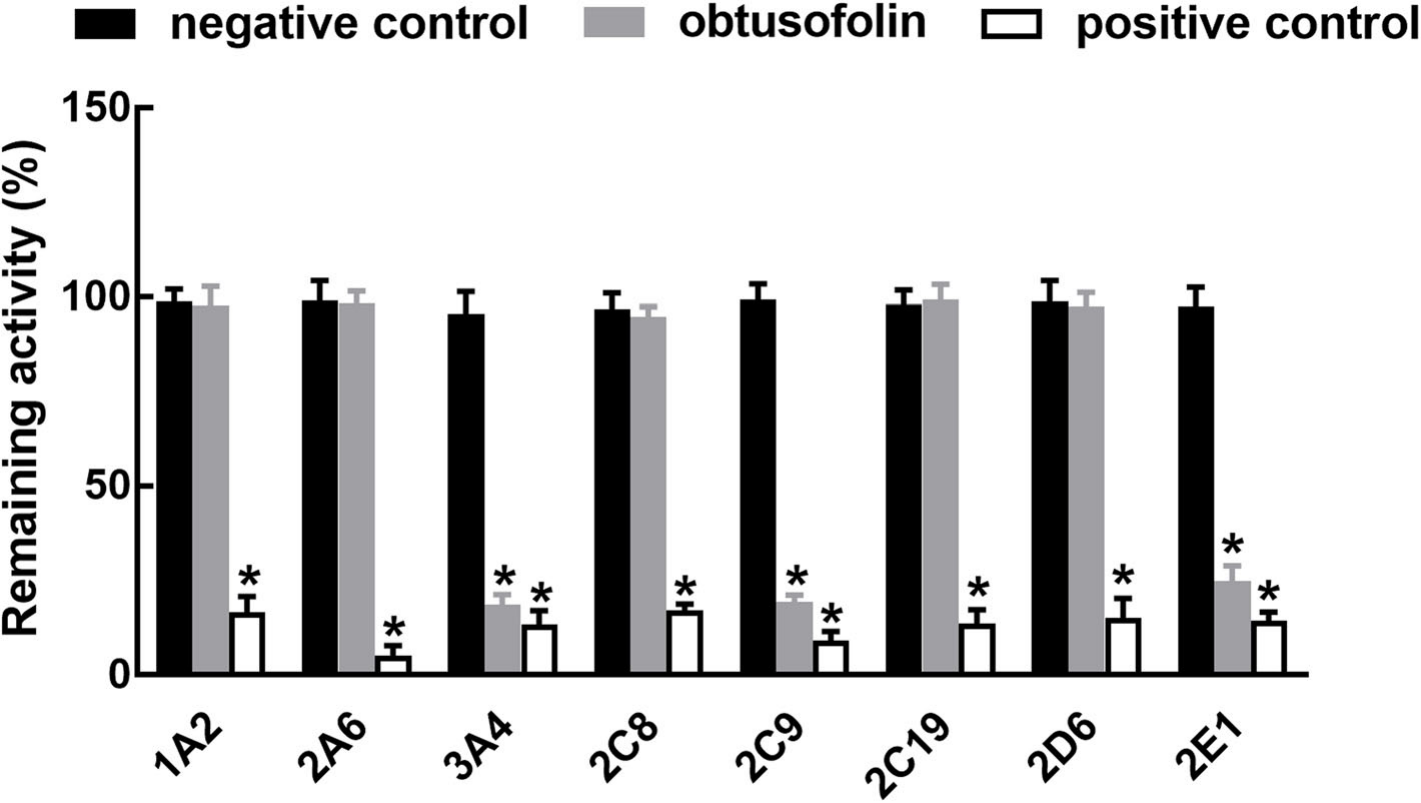
The activity of eight major CYP isoforms in the presence of obtusofolin or positive inhibitors. All CYP isoforms were inhibited by their positive inhibitors. Obtusofolin significantly inhibited the activity of CYP3A4, 2C9, and 2E1. *P < 0.05 relative the negative controls

Corresponding inhibitors dramatically reduced the activity of all CYP isoforms (*P* < 0.05, Fig. 1). Additionally, the activity of CYP3A4, 2C9, and 2E1 was significantly suppressed by obtusofolin in pooled HLMs (*P* < 0.05, Fig. 1). The characteristics of the inhibitory effect of obtusofolin were further evaluated. In the presence of different concentrations of obtusofolin, the activity of CYP3A4, 2C9, and 2E1 decreased with the increase of obtusofolin concentration, indicating the dose-dependent manner of the inhibition of these CYP450s. The IC_50_ values of CYP3A4, 2C9, and 2E1 were obtained as 17.1 ± 0.25, 10.8 ± 0.13, and 15.5 ± 0.16 μM, respectively.

### Obtusofolin acted as a competitive inhibitor of CYP2C9 and 2E1 and a non-competitive inhibitor of CYP3A4

**Fig. 2:**
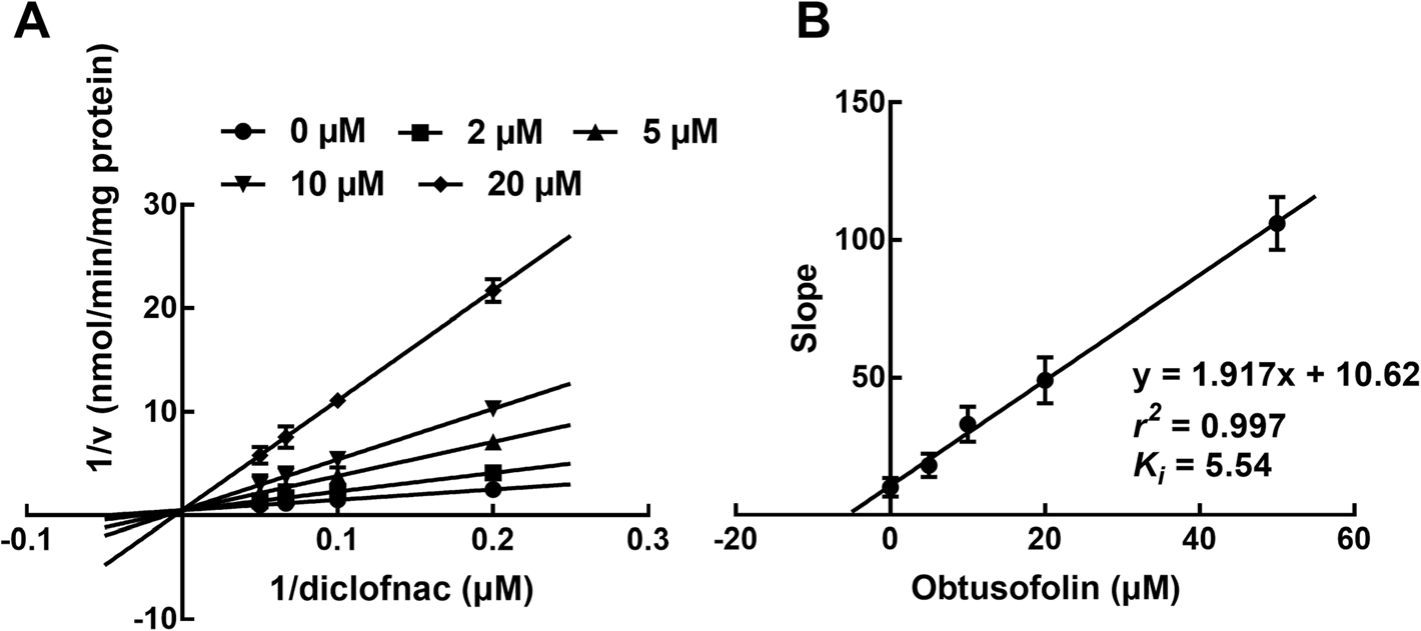
Lineweaver-Burk plots (**A**) and the secondary plot for *K*_*i*_ (**B**) of the inhibition of obtusofolin on CYP2C9 in pooled HLMs. Data were obtained from the incubation with 5–20 μM diclofenac in the presence of 0, 2, 5, 10, and 20 μM obtusofolin

**Fig. 3:**
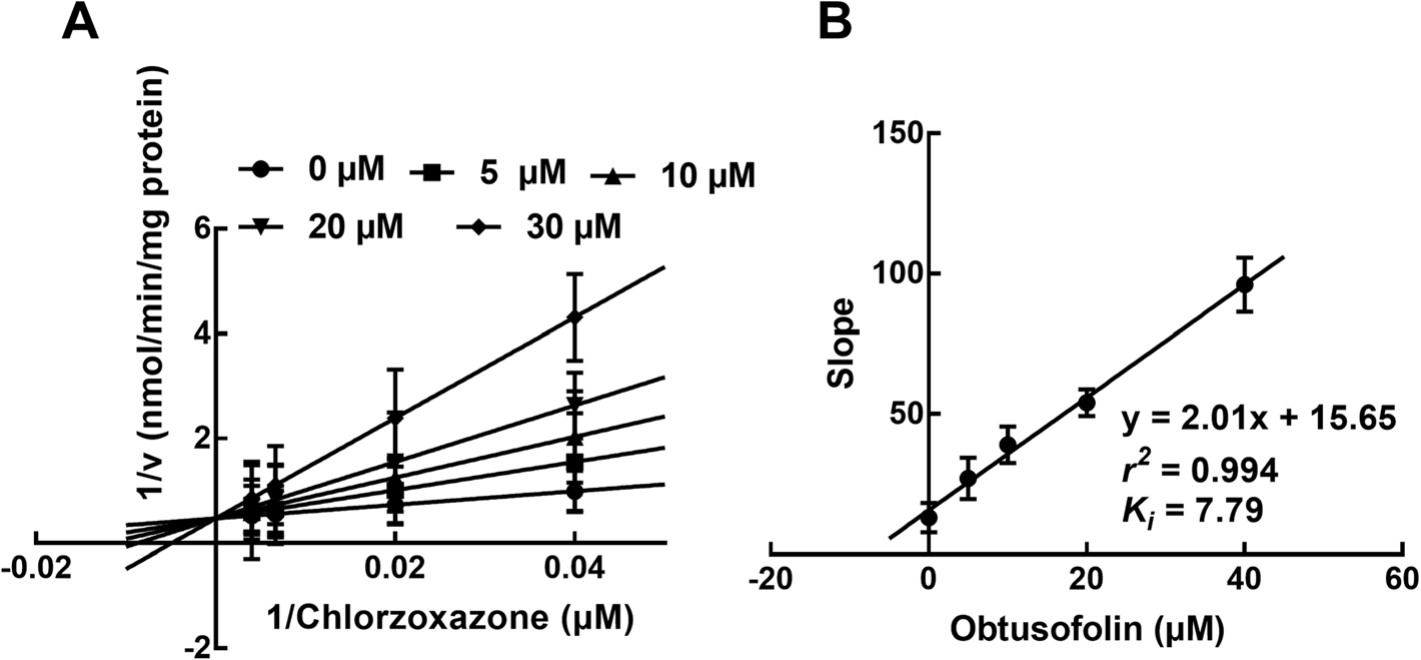
Lineweaver-Burk plots (**A**) and the secondary plot for *K*_*i*_ (**B**) of the inhibition of obtusofolin on CYP2E1 in pooled HLMs. Data were obtained from the incubation with 25–250 μM chlorzoxazone in the presence of 0, 5, 10, 20, and 30 μM obtusofolin

**Fig. 4:**
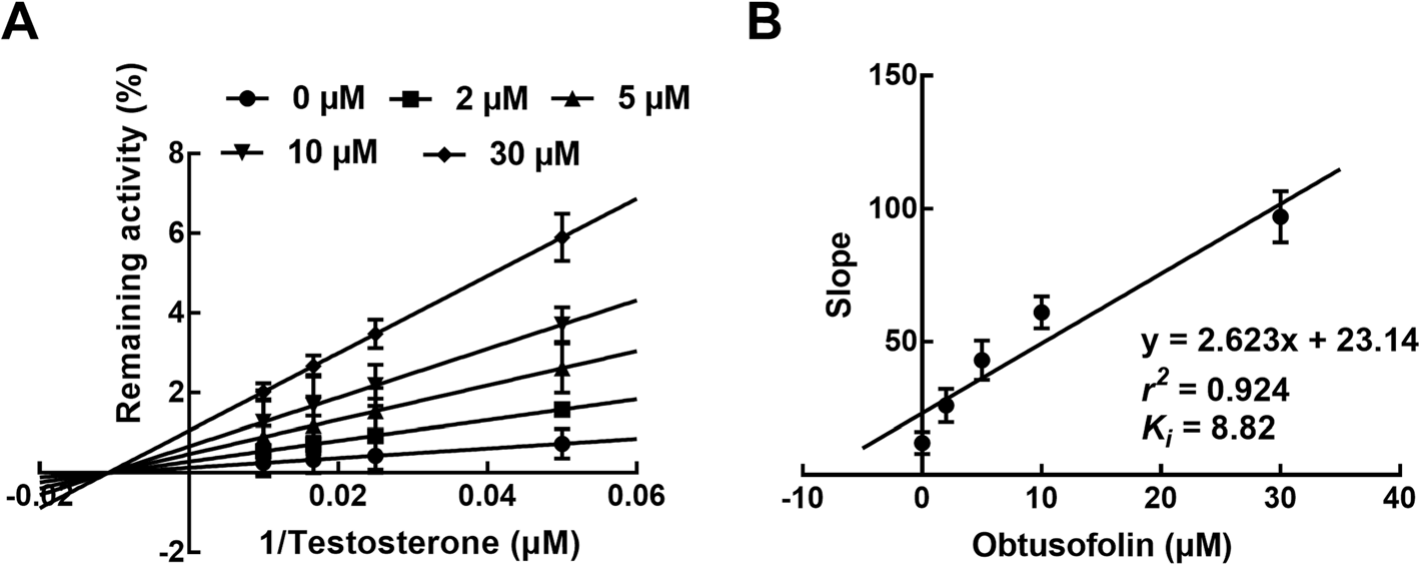
Lineweaver-Burk plots (**A**) and the secondary plot for *K*_*i*_ (**B**) of the inhibition of obtusofolin on CYP3A4 in pooled HLMs. Data were obtained from the incubation with 20–100 μM testosterone in the presence of 0, 2, 5, 10, and 30 μM obtusofolin

In the presence of various substrates and obtusofolin, the inhibition of CYP2C9 and 2E1 was best fitted with the competitive inhibition model with the *K*_*i*_ values of 5.54 and 7.79 μM, respectively (Figs. 2 and 3). While the inhibition of CYP3A4 was best fitted with the non-competitive model with the *Ki* value of 8.82 μM (Fig. 4A and B).

### Obtusofolin inhibited the activity of CYP3A4 in a time-dependent manner

**Fig. 5:**
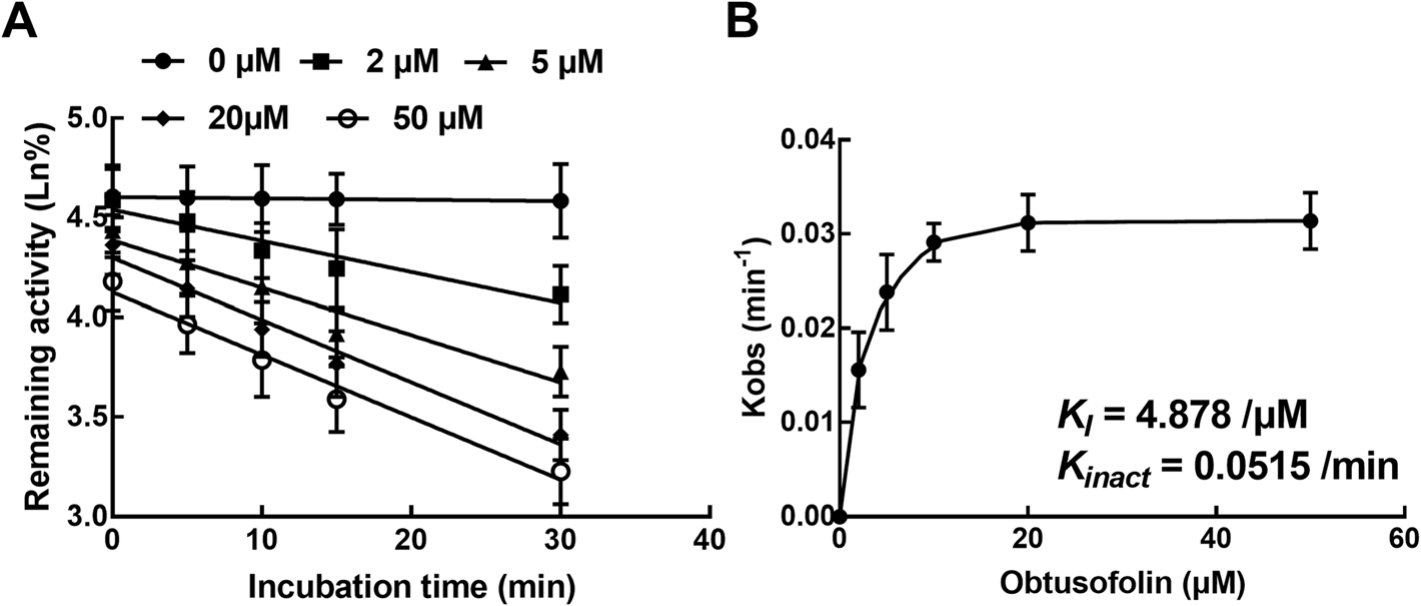
Obtusofolin inhibited the activity of CYP3A4 in a time-dependent manner. **A** Linear regression analysis on the activity versus incubation time in the presence of 0, 2, 5, 20, and 50 μM obtusofolin. **B** Non-linear analysis on the initial rate constant versus the concentration of obtusofolin to obtain the value of K_*I*_ and K_*inact*_

The inhibitory effect of obtusofolin on the activity of CYP3A4 increased with the incubation time (from 5 to 30 min), whereas the inhibitory effect on CYP2C9 and 2E1 was not affected. Furthermore, the time-dependent manner was characterized in the presence of various obtusofolin concentrations. During the time-dependent inhibition of CYP3A4 by obtusofolin, the *K*_*I*_ value was obtained as 4.878 μM^− 1^ and the *K*_*inact*_ value was obtained as 0.0515 min^− 1^ (Fig. 5A and B).

## Discussion

The interaction between CYP450s and various drugs has drawn special attention in the past decades. Numerous evidence has revealed a number of drugs that affected the activity of CYP450s and induced adverse interactions [13–16]. Obtusofolin is the main extraction of *Catsia tora L.*, which has been widely applied in the ophthalmology prescription in the clinic [17]. Obtusofolin has also been demonstrated to possess various pharmacological effects making its clinical use more widely. The influence of obtusofolin on the activity of CYP450s is a critical factor that can provide a reference for the clinical application of obtusofolin.

**Fig. 6:**
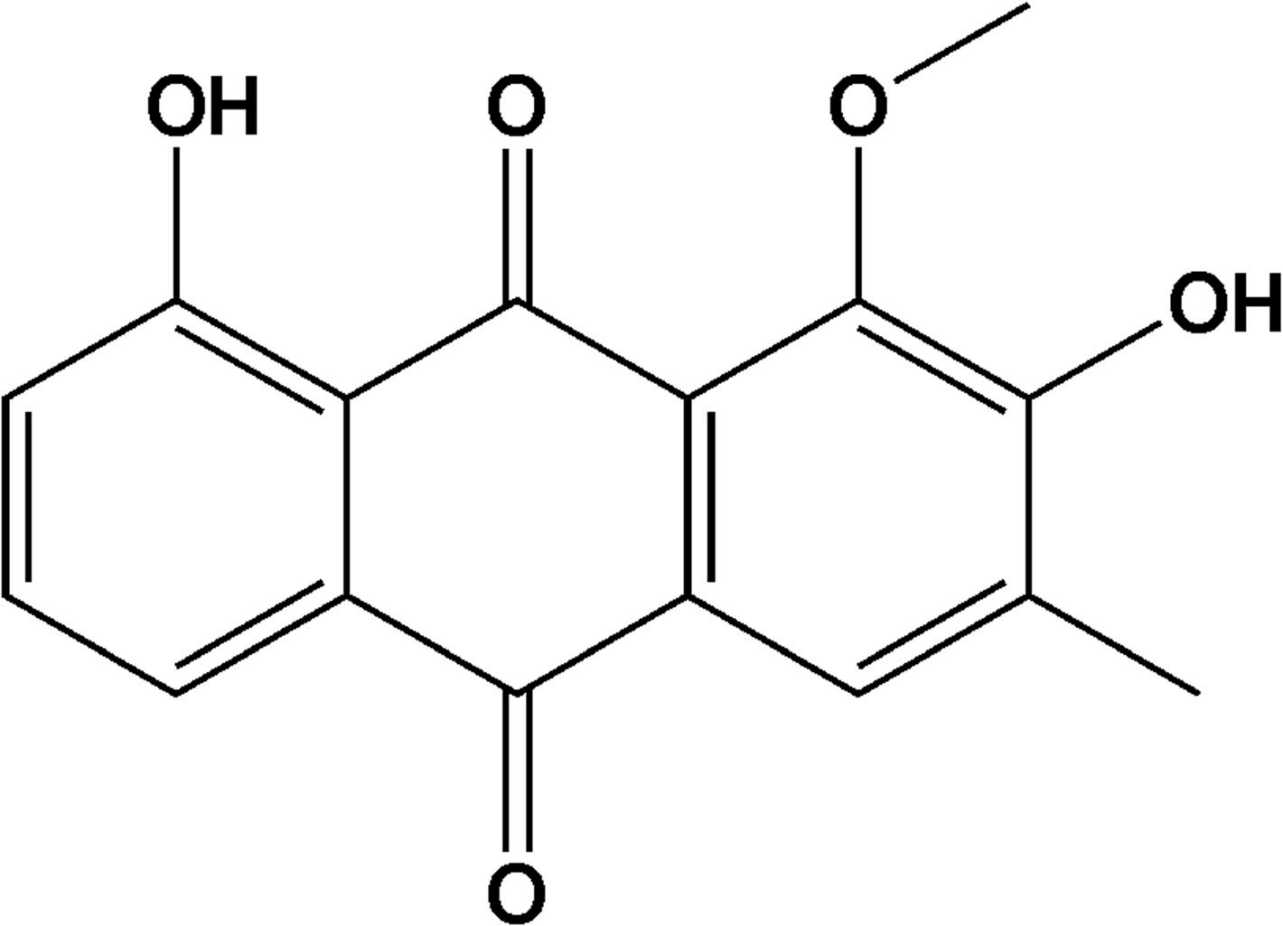
The chemical structure of obtusofolin

Here, obtusofolin was found to inhibit the activity of CYP3A4, 2C9, and 2E1 in the present study. These CYPs were involved in the metabolism of a large number of drugs, the inhibitory effect of obtusofolin, therefore, implied the potential drug-drug interaction [18]. Previously, the inhibition of CYPs has been considered as the main cause during the interaction between various drugs. For example, the inhibitory effect of verapamil on the activity of CYP3A4 has been reported to result in the increased systemic exposure of diverse drugs, such as oridonin and hydroxycamptothecin, which were metabolized by CYP3A4 [19, 20]. Except CYP3A4, CYP2C9 and 2E1 also play vital roles in the pharmacokinetics of assorted drugs. The co-administration of capecitabine and celecoxib, a substrate of CYP2C9, results in a drug-drug interaction, where the maximum plasma concentration and area under the concentration-time curve of celecoxib increased [21]. The inhibition of CYP3A4, 2C9, and 2E1 by obtusofolin was fitted in different models. Obtusofolin served as a non-competitive inhibitor of CYP3A4 and a competitive inhibitor of CYP2C9 and 2E1. This difference in the inhibition model may result from the chemical structure of obtusofolin. The similar structure between obtusofolin and substrates of CYP2C9 and 2E1 may lead to the competition in binding sites, making it the major cause of the competitive inhibitory effect of obtusofolin. Additionally, the inhibition of CYP3A4 was time-dependent. The obtained ratio of KI/Kinact of CYP3A4 indicated that approximately 5.15% CYP3A4 was inactivated per minute in the presence of a saturating concentration of obtusofolin. Kalgutkar et al. [22] reported that aromatic functional groups may be a vital factor responsible for the time-dependent characteristic of chemical compounds, which are included in obtusofolin (Fig. 6).

In previous studies focused on the pharmacokinetic profile of obtusofolin, the maximum of 1.3 mg/kg obtusofolin in rats was 152.5 ± 62.3 ng/mL, which is much less than the IC_50_ values of obtusofolin in the inhibition of CYP3A4, 2C9, and 2E1 [23], indicating the weak possibility of the inhibition of obtusofolin. However, in vivo investigations are needed in further studies to estimate the potential interaction of obtusofolin with CYP450s or drugs metabolized by CYP3A4, 2C9, and 2E1. Additionally, CYP450s are also critical metabolic enzymes in gut. Therefore, the interaction between obtusofolin and CYP450s in gut should attract attention. Furthermore, the interaction between obtusofolin and CYP450s might be different forms in various sourced microsomes. Therefore, more pools of microsomes from other sources should be used in future investigations.

Taken together, obtusofolin was identified as a competitive inhibitor of CYP2C9 and 2E1, and a non-competitive inhibitor of CYP3A4. The inhibition of these CYPs was conducted in a dose-dependent manner with various IC_50_ values, and the incubation time is an important impactor during the inhibition of CYP3A4. The inhibitory effect of obtusofolin implying the potential drug-drug interaction between obtusofolin and drugs metabolized by these CYPs, which needs further in vivo validations.

## Data Availability

The datasets used and/or analysed during the current study are available from the corresponding author on reasonable request.
